# Scrotal Involvement with Testicular Nonseminomatous Germ Cell Tumour

**DOI:** 10.1155/2016/5471862

**Published:** 2016-10-17

**Authors:** C. G. O'Leary, J. A. Allen, F. O'Brien, A. Tuthill, D. G. Power

**Affiliations:** ^1^Department of Medical Oncology, Cork University Hospital, Cork, Ireland; ^2^Temple Street Children's University Hospital, Dublin 1, Ireland; ^3^Department of Urology, Cork University Hospital, Cork, Ireland; ^4^Department of Endocrinology, Cork University Hospital, Cork, Ireland

## Abstract

A 37-year-old male presented with a traumatic injury to the scrotal region necessitating emergency surgery. Evacuation of a haematoma and bilateral orchidectomy were performed. A left sided nonseminomatous germ cell tumour (NSGCT), predominantly yolk sac, was identified. Microscopic margins were positive for tumour. Initial tumour markers revealed an AFP of 22,854 ng/mL, HCG of <1 mIU/mL, and LDH of 463 IU/L. Eight weeks after surgery, AFP levels remained elevated at 11,646 ng/mL. Computed tomography (CT) scanning demonstrated left inguinal adenopathy, 1.5 cm in max dimension. On review, extensive evidence of scrotal involvement was evident. His tumour was staged as stage IIIC, poor risk NSGCT. He was treated with 4 cycles of bleomycin, etoposide, and cisplatin over a 12-week period. His tumour markers normalised after 3 cycles. There was a marked improvement noted clinically. Follow-up CT scans demonstrated complete resolution of his tumour. He later underwent further surgery to remove a small amount of remaining spermatic cord. Histology revealed no malignant tissue. The patient suffered many complications including testosterone deficiency, osteopenia, infertility, and psychological distress.* Discussion*. A small proportion of testicular cancer may present in an atypical manner. The scrotum and testicle have markedly different embryonic origins and therefore a distinct anatomic separation. As a result the scrotum is not a typical site of spread of testicular cancer. Case reports have been described that were managed in a similar manner with good outcomes. Therefore, even with significant scrotal involvement, if timely and appropriate treatment is administered, complete resolution of the tumour may be achieved.

## 1. Case Report

A previously well, 37-year-old farmer presented after sustaining with a major traumatic injury to the scrotal region. This occurred as a consequence of a kick in the groin from a bull while he was tending to his livestock. Physical examination revealed extensive swelling and tenderness of the scrotum. He underwent emergency surgery for evacuation of a haematoma and debridement of necrotic tissue. He required a bilateral orchidectomy as the testicles were split at the time of the trauma. A scrotal approach to surgery was adopted. Unexpectedly, histology identified a left testicular pT4 nonseminomatous germ cell tumour (NSGCT), predominantly yolk sac with lymphovascular invasion. Scrotal and spermatic cord involvement was present. Resection margins were positive for residual microscopic tumour. Presurgery tumour markers, retrospectively analysed serum samples, revealed an alpha-fetoprotein (AFP) of 22854 ng/mL, human chorionic gonadotropin (HCG) of <1 mIU/mL, and LDH of 473 IU/L (10–250 IU/L).

**Figure 1:**
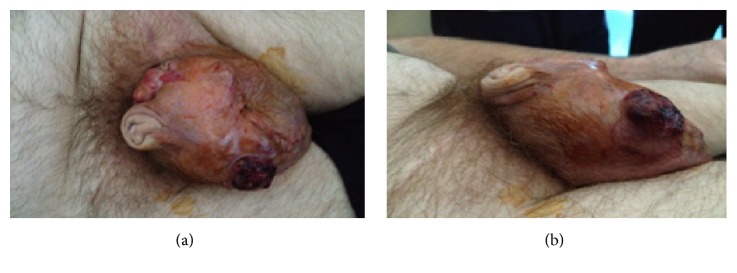
Lateral and anterior views of pretreatment scrotal involvement by nonseminomatous germ cell tumour.

At 8-week follow-up after surgery the AFP level was noted to have remained significantly elevated at 11,646 ng/mL. The patient reported an increase in size of the scrotum in the postoperative weeks. Examination was suggestive of extensive scrotal involvement (Figures 1(a) and 1(b)). A firm swelling, with ulceration and marked discolouration, was noted throughout the scrotum. Tenderness and discharge were evident on palpation of the affected areas. These findings were consistent with disease progression and scrotal contamination by testicular tumour. A pretreatment staging computed tomography (CT) scan of thorax, abdomen, and pelvis demonstrated left inguinal adenopathy, 1.5 cm in maximum dimension.

**Figure 2:**
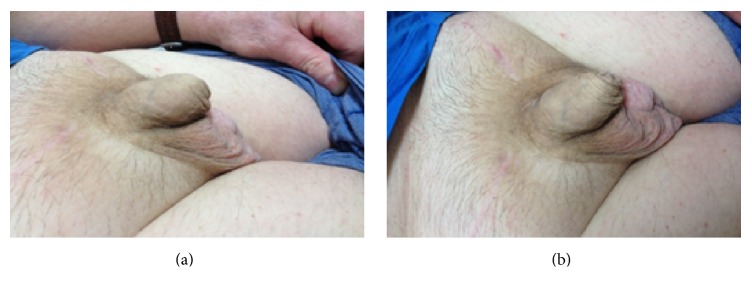
Lateral and anterior views of the scrotum after BEP chemotherapy.

The tumour was classified as stage IIIC, poor risk NSGCT [1, 2]. The patient was commenced on a combination of bleomycin, etoposide, and cisplatin (BEP) chemotherapy. He completed 4 cycles over a 12-week period without complications. His tumour markers demonstrated a constant downward trend with complete normalisation by the end of his third cycle of chemotherapy. Posttreatment imaging confirmed complete response with no further evidence of the left inguinal adenopathy. Additionally there was a marked improvement noted clinically (Figures 2(a) and 2(b)).

Given the incomplete resection he underwent further surgical intervention, by means of an inguinal approach, to remove a small amount of remaining spermatic cord after completing his chemotherapy. Histology on this occasion showed no malignant tissue. To date, 3.5 years out from chemotherapy, the patient remains in remission based on surveillance imaging, tumour marker monitoring, and physical examination.

A number of other issues emerged from this case. These included lifelong testosterone deficiency necessitating supplementation under the supervision of an endocrinologist. A routine Dual-Energy X-Ray Absorptiometry (DEXA) scan identified osteopenia with a* T* score of −1.7. He was commenced on calcium and vitamin D supplementation as well as bisphosphonate therapy. Due to the urgent nature of his presentation sperm banking was not possible. As he was rendered infertile by the surgery he suffered significant psychological distress as he could not father children. This required referral to a clinical psychologist for evaluation and treatment.

## 2. Discussion

Testicular cancer is one of the most treatable solid tumours. The peak incidence is among young men between the ages of 20–40 years. The 5-year survival for nonseminomatous germ cell tumours can be stratified by risk: good risk 91%; intermediate risk 79%; poor risk 48% [1]. Treatment may involve surgery alone or a combination of surgery and chemotherapy. This is dependent on the tumour staging and risk. An inguinal approach to orchidectomy is the recommended standard of care [3]. Deviations from the classic inguinal approach, whilst not recommended, have not been shown to adversely affect outcome [4]. Surveillance with cross-sectional radiology and tumour markers as well as physical exam is recommended after orchidectomy for testicular confined disease in order to identify recurrence early and allow for further intervention [5].

The embryonic origins of the testicle and scrotum are different. The testicles develop from the mesonephros at the 10th thoracic level [6]. They migrate downwards through the inguinal canal, preceded by the gubernaculum, to eventually lie within the scrotum. The male external genitalia originate from the labioscrotal swelling in the fourth month of embryogenesis [7]. Based on this embryonic distinction the testicles and scrotum have separate lymphatic and blood supplies. The drainage system of the scrotum links to the lymphatics of the inguinal region and lower extremities. The main direction of spread of testicular cancer is via the lymphatic system to the retroperitoneum and the para-aortic lymph nodes. Spread via the vascular system to involve distant organs is also possible. Testicular cancer generally does not involve the scrotum as a result of this anatomical separation.

Scrotal involvement from testicular cancer is rare. It has, however, been described previously and is included in the staging of testicular cancer [2]. The optimal strategy for this situation is not well described as there is a lack of prospective randomised trials. Only a few case reports are described. In each case the patient was treated with bleomycin, etoposide, and cisplatin chemotherapy with complete response seen, as in our case [8, 9]. The likely mechanism for scrotal involvement is felt to relate to disruption of the scrotal membranes and therefore seeding of testicular cancer. In the case we report, this required an external insult which would allow for transmission of cancer cells locally; however, spontaneous scrotal involvement can also occur in case of very extensive tumours [8, 9]. As we have outlined treating the patient with 4 cycles of BEP chemotherapy resulted in a dramatic and sustained response. Of note, a pathological complete response was present after histological examination of the residual positive margins once removed after chemotherapy. A second look procedure was carried out in one of the referenced case reports and similarly no residual malignancy was identified after chemotherapy [8]. The logic of resection is that some literature reports identified the cord remnant and hemiscrotum as sanctuary sites for disease and they are therefore less sensitive to chemotherapy [10]. However, historical series have shown no significant differences in outcome between contaminated and cohort series [11, 12].

Given the rarity in this condition we feel this case outlines a reasonable approach to managing an individual who has confirmed scrotal involvement. Patients with proven scrotal involvement from germ cell tumour should be followed up on a regular basis as per the established guidelines for testicular cancer [5].
